# Mogamulizumab-Associated Myositis With and Without Myasthenia Gravis and/or Myocarditis in Patients With T-Cell Lymphoma

**DOI:** 10.1093/oncolo/oyad155

**Published:** 2023-06-07

**Authors:** Cesar A Virgen, Jeffrey A Sparks, Anju Nohria, Meabh J O’Hare, Amrita Goyal, Jordan T Said, Marianne Tawa, Nicole R LeBoeuf, Thomas S Kupper, David C Fisher, Cecilia Larocca

**Affiliations:** Harvard Medical School, Boston, MA, USA; Department of Dermatology, Brigham and Women’s Hospital, Boston, MA, USA; Center for Cutaneous Oncology, Dana-Farber Cancer Institute, Boston, MA, USA; Harvard Medical School, Boston, MA, USA; Department of Medicine, Division of Rheumatology, Inflammation, and Immunity, Brigham and Women’s Hospital, Boston, MA, USA; Harvard Medical School, Boston, MA, USA; Department of Medicine, Division of Cardiovascular Medicine, Brigham and Women’s Hospital, Boston, MA, USA; Harvard Medical School, Boston, MA, USA; Department of Neurology, Brigham and Women’s Hospital, Boston, MA, USA; Department of Dermatology, University of Minnesota, Minneapolis, MN, USA; Harvard Medical School, Boston, MA, USA; Department of Medicine, Brigham and Women’s Hospital, Boston, MA, USA; Center for Cutaneous Oncology, Dana-Farber Cancer Institute, Boston, MA, USA; Harvard Medical School, Boston, MA, USA; Department of Dermatology, Brigham and Women’s Hospital, Boston, MA, USA; Center for Cutaneous Oncology, Dana-Farber Cancer Institute, Boston, MA, USA; Harvard Medical School, Boston, MA, USA; Department of Dermatology, Brigham and Women’s Hospital, Boston, MA, USA; Center for Cutaneous Oncology, Dana-Farber Cancer Institute, Boston, MA, USA; Harvard Medical School, Boston, MA, USA; Center for Cutaneous Oncology, Dana-Farber Cancer Institute, Boston, MA, USA; Department of Medical Oncology, Dana-Farber Cancer Institute, Boston, MA, USA; Harvard Medical School, Boston, MA, USA; Department of Dermatology, Brigham and Women’s Hospital, Boston, MA, USA; Center for Cutaneous Oncology, Dana-Farber Cancer Institute, Boston, MA, USA

**Keywords:** lymphoma, T cell, cutaneous, mogamulizumab, myasthenia gravis, myocarditis, myositis, Sezary syndrome

## Abstract

Mogamulizumab is being increasingly prescribed for the treatment of T-cell lymphomas (MF/SS/ATLL). We conducted a retrospective cohort study to identify muscular immune-related adverse events (irAEs) associated with mogamulizumab in patients with T-cell lymphoma followed at Dana-Farber Cancer Institute from January 2015 to June 2022. We identified 5 cases of mogamulizumab-associated myositis and/or myocarditis (MAM/Mc), 2 additionally affected by myasthenia gravis, among 42 patients with T-cell lymphoma. Three cases experienced ­mogamulizumab-associated rash (MAR) prior to developing MAM/Mc. The incidence (*n* = 5/42, 11.9%) of muscular mogamulizumab-associated irAEs may be higher than has been previously reported in clinical trials and may be of late onset (a median of 5 cycles and as late as 100 days from the last infusion). We highlight the utility of IVIG, together with systemic corticosteroids, for the treatment of these potentially fatal side effects associated with mogamulizumab therapy.

## Teaser

Mogamulizumab is being increasingly prescribed for the treatment of T-cell lymphomas. This brief communication reports on muscular and neuromuscular immune-related adverse events associated with this treatment.

Mogamulizumab is a C–C chemokine receptor type 4 (CCR4)-directed monoclonal antibody used for the treatment of relapsed/refractory mycosis fungoides (MF) or Sézary syndrome (SS), and adult T-cell leukemia/lymphoma (ATLL). From the MAVORIC trial, a fatal event polymyositis and rare reports of myocarditis have been identified.^1^ Given the serious nature of these immune-related adverse event (irAE), we sought to identify the incidence, clinical characteristics, and long-term outcomes of patients with T-cell lymphoma who developed myositis and/or myocarditis.

After approval by the Mass General Brigham Institutional Review Board, including waiver for informed consent given retrospective deidentified data, we performed a search of the Partners Research Patient Data Registry (January 2015-2022; Brigham and Women’s Hospital) for patients who received at least one dose of mogamulizumab and had a diagnosis of T-cell lymphoma were included. Data cutoff was June 2022.

After exclusion of duplicate entries and those who never received mogamulizumab, we identified 5 cases of ­mogamulizumab-associated myositis and/or myocarditis (MAM/Mc) out of 42 T-cell lymphoma patients (38 = MF/SS, 4 = ATLL) followed at our institution (Table 1): case 1, a ­72-year-old female with Sézary syndrome; case 2, an ­89-year-old-male with leukemic mycosis fungoides; case 3, a 73-year-old-male with Sézary syndrome; case 4, a ­56-year-old-female with mycosis fungoides; and case 5, a 65-year-old-female with ATLL (chronic type, with blood, and cutaneous involvement). Of these, 2 patients (cases 1 and 5) also developed ­seropositive myasthenia gravis (MG) and 3 patients (cases 1, 2, and 3) developed a ­mogamulizumab-associated rash (MAR) (Supplementary Fig. S1). Among cases of MAM/Mc, median of follow-up since initiation of mogamulizumab was 18 months (range: 9-42 months). The median onset of MAM/Mc was 168 days (range: 133-241 days) and occurred after a median of 5 cycles (range: 4-6 cycles) of mogamulizumab. In 3 cases (cases 2, 3, and 5), MAM/Mc developed well after discontinuation of mogamulizumab, onset 50-100 days from last infusion. Statins were taken for at least 2 years prior to MAM/Mc in 3 cases (cases 2, 3, and 5). No cases received messenger RNA-based SARS-CoV-2 vaccination within 30 days of MAM/Mc symptom onset (Table 1). No cases reported prior history of autoimmune conditions.

**Table 1.** Demographic characteristics, clinical presentation, and treatment course of each of the cases.

Case	1	2	3	4	5
Diagnosis	SS	Leukemic MF	SS	MF	ATLL
Sex	F	M	M	F	F
Age at treatment, y	72	89	73	56	65
Race	White	White	Black	White	Black
ECOG (baseline)	0	0	0	0	0
Statin use	No	Yes	Yes	No	Yes
Cycles of mogamulizumab (completed)	5	4	5	5	6^a^
Time since last infusion (days)	7	60	50	5	100
MAR by CTCAE (histology, onset)	Maculo-papular, grade 3 (lichenoid, day 96)	Eczema, grade 2 (spongiosis and psoriasiform, day 65)	Erythroderma, grade 3 (psoriasiform, day 119)	—	—
Muscular/neuromuscular toxicity	Myositis; seropositive myasthenia gravis; Myocarditis	Myositis	Myositis	Myocarditis	Myositis; seropositive myasthenia gravis
Preceding illness or vaccination with respect to onset of MAM/Mc (day 0), viral screen	n/a	Influenza vaccine (−152 days), SARS-CoV 2 PCR (−)	COVID-19 vaccine, 1st, 2nd dose (−112, −96 days); influenza vaccine (−220 days), SARS-CoV 2 PCR (−)	COVID-19 vaccine 1st, 3rd doses (−109, −39 days), influenza vaccine (−61 days), microbial screen (−)^b^	COVID-19 vaccine 1st, 3rd doses (−416, −180 days), influenza vaccine (−158 days), SARS-CoV 2 PCR (−)
Seasonality of MAM/Mc, years	Spring 2019	Spring 2020	Summer 2021	Winter 2021	Spring 2022
Onset of MAM/Mc following MOGA initiation (days)	133	157	223	168	241
Clinical findings	Fever; weakness of bulbar, proximal, axial, and respiratory muscles; Takotsubo cardiomyopathy	Bulbar weakness, proximal weakness	No muscle weakness on examination	Hypotension	Proximal limb weakness; fluctuating ophthalmoparesis; proptosis; tachycardia and mild hypertension
Symptoms	Fatigue; muscle cramps; generalized muscle weakness; dysphagia; dropped head; and dyspnea at rest	Fatigue; generalized muscle weakness; dysphagia; dyspnea on exertion	Fatigue; muscle spasms; generalized muscle weakness; peripheral edema	Epigastric pain; Dyspnea; Syncopal episodes	Fatigue; myalgias; generalized muscle weakness; diplopia
Treatment of myositis	IV methylprednisolone: 1 g qd × 3 d, IVIG (1 g/kg) × 1; IV methylprednisolone taper started at 80 mg qd	Oral prednisone 100 mg qd × 10 d, IVIG (0.5 g/kg) × 1; oral prednisone taper started at 60 mg qd	Oral prednisone taper started at 100 mg qd, IVIG (1g/kg) × 1; flare: oral prednisone taper started at 30 mg qd	IV methylprednisolone 1 g qd × 5 d, IVIG (1 mg/kg) × 1; oral prednisone taper started at 80 mg	IV methylprednisolone 1 g qd × 3 d; IVIG (1g/kg) × 1; oral prednisone taper started at 80 mg
Length of time on corticosteroid equivalent to prednisone >10 mg, days	103	200	123	161+ (currently tapering down)	85+ (currently tapering down)
T-cell lymphoma response to mogamulizumab^c^	Global CR	Global CR	Global PR, (due to nodal disease), CR in skin and blood	Global SD	CR

^a^Given complete ATLL remission, mogamulizumab frequency was decreased to every 28 days in cycles 5 and 6.

^b^Microbial screen: adenovirus, coronavirus HKU1, 229E, OC43, human metapneumovirus, human rhinovirus/enterovirus, influenza A/B, parainfluenza virus 1-4, RSV, *Bordetella parapertussis*, *Bordetella pertussis*, *Chlamydia pneumoniae*, *Mycoplasma pneumoniae*, SARS-CoV-2.

^c^The Olsen criteria was used to assess MF/SS response. The Tsukasaki criteria was used to assess ATLL response.

Diagnostic evaluation for MAM/Mc involved laboratory analysis, imaging, functional studies, and muscle biopsy when possible (Table 2). Targeted magnetic resonance imaging (MRI) of the femur in case 1 revealed symmetrical inflammation of proximal muscles. In contrast, whole body positron emission tomography/computed tomography, obtained for restaging of lymphoma, revealed a patchy and ­multi-focal pattern of muscle inflammation in case 2 (Supplementary Fig. S1). Of the 3 patients (cases 1, 2, and 5) that underwent muscle biopsy, only those with image-directed sampling of the muscle identified inflammation (Supplementary Fig. S1). Cardiac MRI was pursued in 2 (cases 4 and 5) of 3 cases with troponin elevation and diagnostic of myocarditis in case 4 (Table 2).

**Table 2.** Diagnostic evaluation performed in cases with suspected myositis and/or myocarditis.

Case	1	2	3	4	5
Laboratory analyses (reference values), not on systemic corticosteroids (peak value)					
AST (10-50 U/L)/ ALT (10-50 U/L)	167/175	50/45	80/72	83/51	271/244
CPK (26-192 U/L)	2396	—	1373	602	5090
Myoglobin (<71.0 ng/mL)	—	—	740.9	—	—
CK-MB (<4.3 ng/mL)	403	—	—	102.8	234
High-sensitivity troponin (<9 ng/L)	1366	—	23	4120	1979
Troponin I (<0.3 ng/mL)	—	—	—	—	1.52
LDH (135-225 U/L)	783	359	344	593	975^a^
ESR (<30 mm/h)/ CRP-hs (<3.0 mg/L)	−	−	44/9.5	5/45.5	40/47.8
NT-proBNP (<900 pg/mL)	26 524	—	—	6069	266
Imaging	MRI femur: Muscle edema of bilateral adductor, iliacus, gluteal musculature, hamstrings, vastus lateralis; subfascial and subcutaneous edema	PET/CT: Extensive FDG avid muscular lesions (SUV max of 3.5-11)	—	Cardiac MRI: concentric pericardial effusion, patchy late gadolinium enhancement (LGE) throughout LV, mainly septal. Diffuse LV myocardial edema	CT C/A/P unremarkable. Cardiac MRI: LV size normal, LVEF 60%, no regional wall motion abnormalities, no evidence of edema or LGE
Electrocardiogram	Inferior infarct (age indeterminant), nonspecific T wave abnormalities in anterior lateral leads	Unchanged from baseline	Unchanged from baseline	ST elevations in V1 and V2	Unchanged from baseline.
Echocardiogram	LVEF 25%, LV diffuse mid-level hypokinesis/akinesis, apical akinesis	—	Unchanged from baseline	LVEF 40%, LV diffusely hypokinetic with regional variation, small pericardial effusion	LVEF 65%, no wall motion abnormalities
CT angiogram; left heart catheterization	RCA occlusion (chronicity unknown)	—	—	Normal	—
Positive serologies	Anti-AChR (0.22 nmol/L), striational Ab (1:1920), AchR modulating Ab (61%); myositis antibody panel^b^ U1RNP (weak), Ro60 (weak)	—	ANA (1:320, speckled)	—	Anti-AChR (0.47 nmol/L); Myositis antibody panel Ro60 (weak)
Negative serologies	Anti-HMGCR, ANA; paraneoplastic panel^c^; ­anti-CRMP5	Comprehensive myositis panel^d^	Anti-AChR	ANA	Anti-HMGCR; ANA
Muscle biopsy	Moderate excess variability in myofibril size, myophagocytosis, admixed T-cell infiltrate surrounding individual myofibers and within endomysium^e^	Polymorphous endomysial infiltrate of histiocytes, lymphocytes, scattered eosinophils	—	—	Skeletal muscle with mild variation in fiber size and scattered atrophic type 2 fibers^f^
Electromyography	Consistent with myopathy with associated muscle membrane irritability. Interpretation of 3 Hz repetitive nerve stimulation limited by movement artefact but showed decremental response in compound motor action potential amplitude (up to −10.7%)	—	—	—	Consistent with myopathy with associated muscle membrane irritability. 3 Hz repetitive nerve stimulation with abnormal decrement in compound motor action potential amplitude (up to −11.9%)

^a^LDH value obtained 2 days after starting systemic corticosteroids.

^b^Myositis antibody panel: Jo-1, PL-7, PL-12, EJ, OJ, PM-SCL, Ku, U1RNP, U2RNP, RO60.

^c^Paraneoplastic panel: anti-neuronal nuclear, ANNA-2, ANNA-3, AGNA-1, PCA-1, PCA-2, PCA-TR, amphiphysin, CRMP-5-IgG, striational antibody, CA Chan Bind PQ & N, ACH receptor binding ab, ACH ganglionic neuronal, neuronal VGKC.

^d^Comprehensive myositis antibody panel: Ro52, Ro60, Smith/RNP, Jo-1, PL-12, PL-7, EJ, OJ, SRP, Ku, PM/SCL-100, U3 RNP, Mi-2, P155/140, TIF-1 gamma, SAE1, MDA5, NXP2.

^e^Obtained after initiation of high dose corticosteroids.

^f^Not image-guided muscle biopsy.

At the time of mogamulizumab discontinuation, 4 cases achieved a global response (complete response in cases 1, 2, and 5; partial response in case 3) (Table 1). The duration of response ranged from 6 to 36 months and ongoing in 2 cases (minimum follow-up of 9 months).

All cases made a complete recovery following treatment with intravenous immunoglobulin (IVIG) and systemic corticosteroids (Table 1). IVIG was added given lack of clinical improvement on high dose systemic corticosteroids in the first 2 cases and was associated with the first signs of ­patient-reported improvement in respiratory strength (case 1) and dysphagia (cases 1 and 2) within 2 days of administration. Given this favorable response, IVIG was added irrespective of response to systemic corticosteroids in subsequent cases at the time of MAM/Mc diagnosis.

We report 5 cases of immune-mediated myositis with and without seropositive myasthenia gravis and/or myocarditis in patients with T-cell lymphoma (MF/SS/ATLL). The most frequent toxicities of mogamulizumab therapy are infusion reactions and cutaneous eruptions.^2,3^ MAM/Mc is reportedly rarer but a potentially fatal toxicity. In a phase II trial, 1/37 patient developed myositis (grade 3) after 7 cycles.^4^ In the MAVORIC trial, 1/184 patient developed MAM after 4.5 months (grade 5); the patient died from respiratory muscle failure and pneumococcal pneumonia.^2,5^ Another MAVORIC patient developed myositis (grade 3) and myocarditis after 5 months.^2,5^ In our center, the incidence of MAM/Mc (*n* = 5/42, 11.9%) is higher than what has previously been reported. Similarly, our 5 cases presented 4-8 months after starting treatment with mogamulizumab. Interestingly, 2 of our cases (cases 2 and 3) had been off mogamulizumab for 1-2 months prior to the onset of MAM/Mc due to extensive MAR. Another patient (case 5) had been off mogamulizumab for 3 months prior to the onset of MAM/Mc with MG due to elevated liver enzymes.

We hypothesize that MAM/Mc is a result of the drug’s effect on regulatory T cells (T_reg_), which also express CCR4.^3^ Mogamulizumab depletes T_regs_ and potentially disinhibits autoreactive T cells, leading to adverse events that mirror T-cell-mediated autoimmune syndromes.^3^ IrAEs observed in MAVORIC include rash, hepatitis, myocarditis, myositis, pneumonitis, polymyalgia rheumatica, and Miller-Fisher syndrome.^2,5^ The incidence and spectrum of ­mogamulizumab-associated irAEs remains poorly characterized as it is currently prescribed much less frequency than immune checkpoint inhibitors (ICIs). However, given its potential to decrease T_reg_ cells, it is being increasingly studied in combination with ICIs across tumor types, which is cause for heightened awareness of these potentially fatal irAEs. Mortality risk is driven by the potential for involvement of respiratory and bulbar muscles, increased risk of infection, and concomitant myocarditis. It is also important to screen for MG (in patients that develop neuromuscular symptoms), which has not been previously reported with mogamulizumab, but frequently overlaps with ICI-induced myositis. We recommend MAM/Mc be treated according to expert consensus guidelines for the management of ICI-myositis.^6^ We highlight the utility of IVIG for MAM/Mc, which parallels the known efficacy of IVIG for ­corticosteroid-refractory dermatomyositis and polymyositis.^7^

Risk factors for the development of MAM/Mc are unknown but may be unique to TCL patients with cutaneous disease. While MAM/Mc patients have a distinct clinicopathologic presentation from dermatomyositis, the presence of active skin inflammation, either due to TCL or MAR, may unmask shared antigens between the skin and muscle tissue. Early data, including our cases, suggests that the induction of these immune-mediated toxicities may correlate with durable complete anti-tumor responses.^8,9^

We report 5 cases of immune-mediated myositis with and without seropositive myasthenia gravis and/or myocarditis in patients with T-cell lymphoma (MF/SS/ATLL) treated with mogamulizumab. IVIG, together with systemic corticosteroids, provided safe and effective control of these potentially fatal toxicities. Future research should explore the risk factors associated with the development of these ­mogamulizumab-associated irAEs, in addition to determining the best treatment strategies to mitigate these irAEs.

## Supplementary Material

oyad155_suppl_Supplementary_Figure_S1

oyad155_suppl_Supplementary_Figure_Caption

## Data Availability

The data underlying this article will be shared on reasonable request to the corresponding author.

## Prior Presentation

Two of the cases in this manuscript were presented at the United States Cutaneous Lymphoma Consortium Annual Virtual Workshop on June 26, 2021.
