# Supplementary figures and images for: Mogamulizumab-Associated Myositis With and Without Myasthenia Gravis and/or Myocarditis in Patients With T-Cell Lymphoma
Source: Oncologist. 2023 Jun 7;28(8):e694–8. doi: 10.1093/oncolo/oyad155 (PMC10400128; doi:10.1093/oncolo/oyad155)

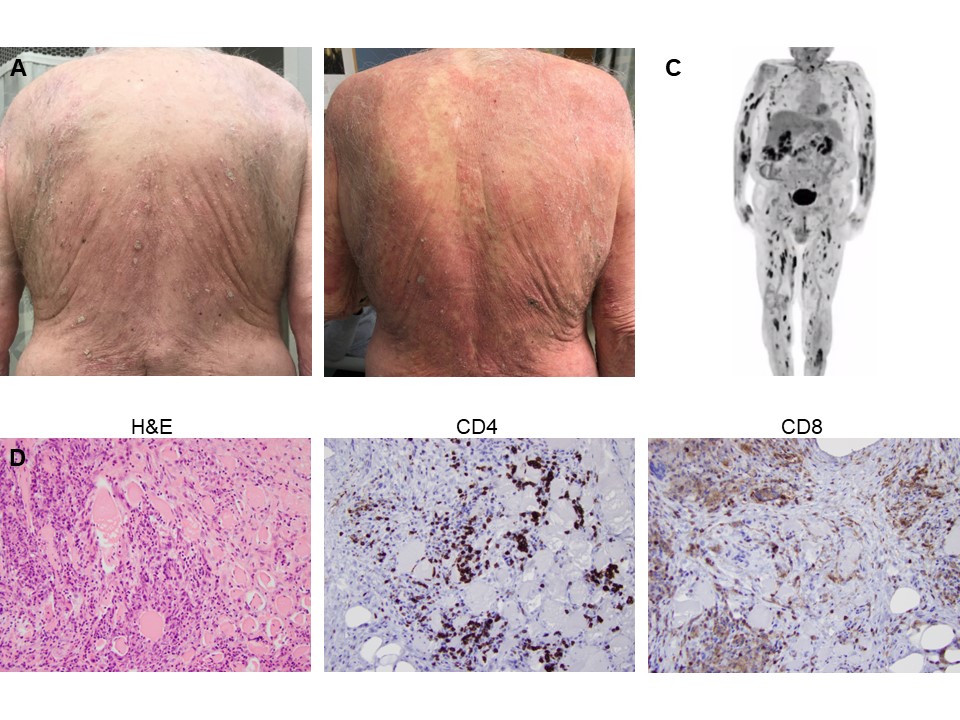

Supplement: oyad155_suppl_Supplementary_Figure_S1 [file oyad155_suppl_supplementary_figure_s1.jpeg]
