# Supplementary material for: Mogamulizumab-Associated Myositis With and Without Myasthenia Gravis and/or Myocarditis in Patients With T-Cell Lymphoma
Source: Oncologist. 2023 Jun 7;28(8):e694–8. doi: 10.1093/oncolo/oyad155 (PMC10400128; doi:10.1093/oncolo/oyad155)
Supplement: oyad155_suppl_Supplementary_Figure_Caption [file oyad155_suppl_supplementary_figure_caption.docx]

**SUPPLEMENTAL FIGURE LEGEND**

**Supplemental Figure 1:** Selected clinical, imaging, and histopathologic findings in case 2. A) Representative clinical photograph (back) at initial diagnosis of mycosis fungoides, showing coalescing eczematous plaques. B) Mogamulizumab-associated rash, representative clinical photograph (back) showing diffuse eczematous patches. C) PET-CT imaging showing numerous FDG-avid intramuscular lesions (SUV max of 3.5-11) scattered throughout the body. D) Image-guided muscle biopsy demonstrating skeletal muscle with polymorphous endomysial infiltrate of histiocytes, small CD4+/ CD8+ lymphocytes (1:1), and scattered eosinophils.
